# Hyperekplexia: A Treatable Seizure Mimicker in Infants

**DOI:** 10.7759/cureus.38082

**Published:** 2023-04-24

**Authors:** Sai Chandar Dudipala, Raja Vijendra Reddy, Roop Shankar

**Affiliations:** 1 Pediatrics, Prathima Institute of Medical Sciences, Karimnagar, IND; 2 Pediatric Neurology, Star Women & Children Hospital, Karimnagar, IND; 3 Pediatrics, Star Women & Children Hospital, Karimnagar, IND

**Keywords:** clonazepam, vigevano manouvre, glra1 gene, epilepsy, hyperekplexia

## Abstract

Hyperekplexia (HK) or startle disease is an uncommon, early infantile onset, potentially treatable neurogenetic disorder. It is characterized by an exaggerated startle reflex in response to tactile or acoustic or visual stimuli followed by generalized hypertonia. It is caused by genetic mutations in a number of different genes such as *GLRA1*, *SLC6A5*, *GLRB*, *GPHN*, and *ARHGEF9*. HK is frequently misdiagnosed as a form of epilepsy and is advised for prolonged antiseizure medications. Here, we report a two-month-old female child with HK, who was treated for epilepsy. Next-generation sequencing revealed a pathogenic homozygous missense mutation of variant c.1259C>A in exon 9 of the *GLRA1 *gene that was compatible with the diagnosis of hyperekplexia-1.

## Introduction

Startle disease or hyperekplexia (HK) is a rare early infantile onset neurogenetic disorder characterized by exaggerated startle response to sudden tactile, auditory, and visual stimuli followed by generalized stiffening of the body [1,2]. In contrast to a physiologic startle response, the excessive startle leads to prolonged stiffening without loss of consciousness [3]. These events are misdiagnosed as seizures and treated with anti-seizure medications [4]. It is caused by mutations in different genes such as *GLRA1*, *SLC6A5*, *GLRB*,* GPHN*, and *ARHGEF9* [5-7].

Here, we report a two-month-old female child with HK, in whom clinical exome sequencing identified a homozygous *GLRA1* gene mutation. The child was well recovered with clonazepam and had a mild motor delay at one year of age.

## Case presentation

A two-month-old female child was born to non-consanguineous parents and presented to the pediatric neurology clinic with complaints of paroxysmal events in the past 10 days of life. These events were characterized by a sudden flexion of all four limbs with jerking for three to five seconds for sudden sounds or touch; those events gradually increased and at the time of presentation, five to six events per day were noticed. Antenatal and birth history were uneventful. At one month of age, the child had been admitted to the neonatal intensive care unit (NICU) with acute life-threatening events (ALTE). This event was characterized by sudden jerky movements of limbs with stiffening of the body followed by cessation of respiration for two to three minutes followed by normal. At that time, the child was evaluated with magnetic resonance imaging (MRI) brain, electroencephalogram (EEG), and blood routines, which were normal. The child was started on levetiracetam and phenobarbital. After these medications, events decreased, but in the past 10 days increased in frequency. 

On examination, there was no dysmorphism, and normal anthropometry was observed. Neurological examination was normal. These events were reproducible with tapping on the tip of the nose or glabella or sudden sound. The child was clinically suspected of HK and advised for clinical exome sequencing. Next-generation sequencing (NGS) detected a pathogenic homozygous missense variation in exon 9 of the *GLRA1* gene that was compatible with the diagnosis of hyperekplexia 1. Family screening was advised, but due to financial constraints, they were unable to do the screening. Levetiracetam and phenobarbital were stopped, and clonazepam was initiated at 0.03 mg/kg/day and gradually increased up to 0.1 mg/kg/day. Parents were counseled about Vigevano's maneuvers during the attack. On follow-up, there were no life-threatening events, but occasional startling events for loud noises present. At present, the child's age is one year, and has a mild developmental delay with rare nocturnal jerks. 

## Discussion

HK is a rare, potentially pharmacoresponsive, non-epileptic neurogenetic condition. The prevalence rate of HK is <1/1000000. Startle reflex is a normal reticular and cortical reflex. It refers to sudden jerky movements of the body in response to various stimuli, which is considered as a protective mechanism [8]. When the startle reflex is exaggerated, it interferes with normal activities, causing life-threatening apnea, and rigidity. The pathological state is termed HK [9]. The hypertonia may be predominantly truncal and attenuated during sleep. Neonates may have prolonged periods of rigidity. The events tend to resolve after infancy, but children may continue to experience nocturnal jerks [10]. In our case too, after one year of age, the child had only nocturnal jerks. 

HK is a heterogeneous genetic disorder. HK is caused by mutations in a number of different genes involved in glycinergic neurotransmission. The genes include *GLRA1, SLC6A5, GLRB, GPHN*, and *ARHGEF9* [5,6]. Thirty percent of HK cases have mutations of *GLRA1* gene [11]. In our case, the child had a homozygous missense mutation in the *GLRA1* gene.

HK is frequently misdiagnosed as a form of epilepsy and treated with two antiseizure medications. Diagnosis of HK is routine with prior awareness of the disease. The exaggerated startle response that can be elicited by nose tapping is pathognomic and included in the examination of suspected patients [12]. Usually, serum electrolytes, neuroimaging, EEG, and other biochemical tests are normal in these HK patients. Video-EEG or EEG with simultaneous observation by an experienced technician may be helpful in the differential diagnosis. Attacks of hypertonicity with cyanosis can be stopped by a simple intervention called the Vigevano maneuver, which consists of flexion of the head and legs towards the trunk [13].

HK is a potentially treatable condition as compared to the majority of neurogenetic disorders. Clonazepam, a GABARA1 agonist, is the most effective drug for HK. Infants usually require high doses (0.1-0.2 mg/kg/day) of clonazepam to decrease the episodes of startling and life-threatening events. Hence, It reduces morbidities and mortalities associated with the disease [14]. In the present case, the child's required maximum dose of clonazepam was 0.1 mg/kg/day.

## Conclusions

HK is a rare non epileptic genetic condition, which is commonly misdiagnosed as epilepsy. Prior awareness of this condition can guide towards diagnosis by genetic sequencing and avoid unnecessary medications.
